# Using government data to understand the use and availability of medicines for hypertension and diabetes: lessons from Peru

**DOI:** 10.1186/s40545-022-00481-5

**Published:** 2022-11-18

**Authors:** Janeth Tenorio-Mucha, María Lazo-Porras, Jessica Zafra, Margaret Ewen, David Beran

**Affiliations:** 1 CRONICAS Centre of Excellence in Chronic Diseases, Universidad Peruana Cayetano Heredia, Lima, Peru; 2 Division of Tropical and Humanitarian Medicine, University of Geneva and Geneva University Hospitals, Rue Gabrielle-Perret-Gentil 4, Geneva, Switzerland; 3 Health Action International, Amsterdam, The Netherlands

**Keywords:** Essential drugs, Drug utilization, Losartan, Metformin, Segmented regression, Time series

## Abstract

**Background:**

Regular measurement of the availability and use of key medicines for non-communicable diseases allows the tracking of progress to achieve equitable access to medicines. Using a country-level public sector monitoring system for medicine supply, we aim to evaluate the availability and use of losartan 50 mg tablets and metformin 850 mg tablets between 2015 and 2020 investigating the impact of different policy changes and the COVID-19 pandemic.

**Methods:**

Data from the Peruvian National System of Medicine Supply were analyzed using an interrupted time series analysis with known and unknown structural breaks. The outcomes assessed were medicine use (monthly doses dispensed at facilities over time) and medicine availability (proportion of facilities that reported having the medicine available).

**Results:**

The use of losartan and metformin at the primary level of care had a linear increasing trend over the period of analysis. In secondary and tertiary levels of care, there were no increases but some significant level and trend changes of doses dispensed at different times between 2017 to 2019, but none were related to the change of procurement procedures. At all levels of care, the COVID-19 onset in April 2020 caused an abrupt drop in doses dispensed especially at the primary level. Regarding availability, we found an increasing linear trend in the primary level of care for both medicines. In secondary and tertiary levels of care, the availability fluctuated between 40 and 95%. The onset of the COVID-19 pandemic did not significantly impact medicine availability, except for losartan in the tertiary level of care.

**Conclusion:**

The availability and proper dispensing of first-line medicines for hypertension and diabetes is an essential factor for sustainable and equitable treatment. Health care systems need to be prepared for forecasting the increasing demand of medicines for chronic diseases, but also to maintain effective medicine supply chains during humanitarian crisis like pandemics.

**Supplementary Information:**

The online version contains supplementary material available at 10.1186/s40545-022-00481-5.

## Background

Worldwide, the number of people with hypertension (HTN) and type 2 diabetes (T2D) has increased dramatically over the last 30 years [1, 2]. This increase in disease burden should be accompanied by an increase in availability and use of medications for the management and control of these conditions [2, 3]. In the Global Action Plan for the prevention and control of non-communicable diseases (NCDs) developed by the World Health Organization (WHO), a voluntary target of an 80% availability of affordable basic technologies and essential medicines is included [4]. However, despite the implementation of different policies to improve access to medicines [5, 6] low availability and poor affordability of essential medicines for NCDs in low- and middle-income countries (LMICs) is widely reported [7–10].

The pattern of medicine utilization is not only affected by the magnitude of the disease, but also by health and pharmaceutical policies and practices [11]. Health policies can either facilitate or hinder access to medicines depending on the health system’s capacity and organization [12], level of financing and expenditure [13], and whether or not insurance schemes are present [13], among others. Pharmaceutical policies and practices involve marketing, regulatory affairs, and the supply chain for medicines [11, 14]. Any change in one or more of these policies or practices may impact the availability, acceptability, affordability, accessibility or quality of medicines [12]. Therefore, it is important to have tools that allow constant monitoring of the pattern of medicine use and availability to track progress on equitable access to treatments for patients.

In Peru, a Latin American country, according to the national pharmaceutical policy, the public health care system should ensure universal access to medicines as an essential component of comprehensive care through rational selection, affordable prices, sustainable financing and reliable supply [15]. Nevertheless, differentiated access to medicines in public or private health facilities, geographic location and level of care (tertiary versus primary) [16–18] has been found. Suboptimal availability of medicines in the public sector such as enalapril, losartan, metformin or insulin for the treatment of common NCDs has been reported in Peru [18–20]. This means that although patients should receive their medications free of charge, they may have significant out-of-pocket-expenses to purchase these in the private sector due to their unavailability in the public sector. Although previous studies suggest poor availability of medication for HTN and T2D, it is not possible to extrapolate these results to the national level nor to explore variations over time in the availability since the methods used in these studies are convenience samples, are cross-sectional studies or include only one level of care [17, 18].

Peru has a nationwide monitoring system for the supply of medicines in the public sector that keep a record of the availability and quantity of medicines used in each health facility. The use of systematic data allows for a stratified analysis by level of care (primary, secondary or tertiary) and/or geographical organization (district, province, or regions). Such a tool could also assess the impact of policies or interventions in the procurement, distribution or prescription of medicines [21]. Therefore, we aimed to test the impact of two changes on the availability and use of metformin and losartan tablets. The first one corresponds to a change in the procurement process for these medicines in Peru and the second to restrictions due to COVID-19. A secondary aim was to identify features and weaknesses of the data collected in the monitoring system to evaluate how this could be further used in research.

## Methods

### Context

In Peru, patients can access medicines in public or private pharmacies. Public pharmacies may belong to institutions of the Ministry of Health (MoH), Social Security or the Armed and Police Forces. The MoH is the main provider of public health services, almost 90% of all health care facilities nationwide belong to the MoH [22]. For MoH facilities, the supply of medicines is organized through national, regional or institutional procurement [23]. National procurement can be done using centralized or decentralized supply. In centralized supply the selection, estimation, and budget come from the central level of the MoH, whereas in decentralized supply, this is performed by regional health departments and hospitals independently. In both cases, the tendering process is managed and supervised by National Center for the Supply of Strategic Health Resources—CENARES (Centro Nacional de Abastecimiento de Recursos Estratégicos en Salud, in Spanish). Once distributed to health facilities, medicines are dispensed to patients who are affiliated to the public health insurance (Seguro Integral de Salud-SIS) free of charge or in exchange for payments to those who are not insured.

### Study design

We used an interrupted time series (ITS) design, a strong quasi-experimental method for the evaluation of interventions in which the investigator does not have control [24]. ITS allows for the immediate and/or over time assessment of the magnitude of the change in an outcome of interest based on an intervention, and whether other factors different to the intervention could explain the change [25]. ITS have been previously used in the area of medicine utilization research, to assess medicine policy changes caused by reimbursement changes, guideline changes, safety advisories, patent expirations among others using in most of the cases administrative data collected at different points in time [26].

In this study, we aimed to examine how availability and use of medicines stratified by level of care that is primary, secondary, and tertiary was impacted by different changes over time. Our analysis is focused on losartan 50 mg tablets and metformin 850 mg tablets, medicines used for the treatment of HTN and T2D, respectively. Both are the first-line medicines prescribed for HTN and T2D according Peruvian clinical guidelines [27, 28].

### Data sources

This study used data from the Peruvian National System of Medicine Supply called SISMED (Sistema Integrado de Suministro de Medicamentos e Insumos Médico-quirúrgicos, in Spanish). The database includes information from all facilities that are administered by the MoH, which represents 92.8% of all health facilities nationwide [29]. The SISMED dataset registers the monthly quantity delivered of all medicines in health facilities, it also contains variables on health care centers such as level of care (primary, secondary, or tertiary). We used monthly data from January 2015 to December 2020 extracted from SISMED. A total of 8315 facilities with existing data were analyzed, there were 8136, 145 and 34 facilities for the primary, secondary and tertiary level of care, respectively.

### Outcomes measures

For this study, the outcomes were availability and use. Medicine use was defined as the monthly doses dispensed at facilities over time. We estimated the aggregated monthly dispensed volume of medicines nationwide and divided by the equivalent monthly pills in daily defined doses (DDDs). According the WHO Collaborating Centre for Drug Statistics Methodology [22], the DDDs for metformin and losartan corresponds to 2 g and 50 mg, respectively, which is equivalent to 71 pills of metformin 850 mg and 30 pills of losartan 50 mg per month.

Medicine availability was defined as the proportion of facilities that reported having the medicine available regardless of the quantity. When a monthly dispensation other than zero was reported, we considered the medicine was available in the facility. Thus, we estimated availability proportion using the number of facilities with medicine available as numerator and total number of facilities per level of care as denominators.

### Structural changes

In 2018 the NCD unit from the MoH decided to centralize procurement for losartan and metformin, with quantification and budget coming directly from the MoH. The centralized purchase was held for the first time in 2018 and the distribution started in 2019 (exact month varies by health care facility). It was posited that through the centralized purchase the availability and utilization of medicines should have increased because budget, quantification and supply issues were addressed plus the MoH coordinated medicine distribution avoiding over or understocks.

The restrictions due to COVID-19 are considered as the second change. The first case of COVID-19 in Peru was reported on March 6 2020, the WHO declared COVID-19 pandemic on March 11 2020, and the Peruvian government declared a national lockdown on March 17, 2020 that lasted until June 30, 2020. From April 2020 the impact of COVID-19 were that many primary care facilities were closed, health care delivery was focused on COVID-19, the delivery of medicines was interrupted or not performed at all in the public sector, and very strict restrictions were placed on the population [30].

### Statistical analysis

Quantity delivered and proportion of availability were plotted over time monthly. The graphs were visually examined to assess trends. Segmented linear regression models to statistically estimate level and trend changes were used. Level changes show an immediate change after an intervention, while trend changes present a modification in the pattern after the intervention. Quantity delivered and proportion of availability were first fitted and then autoregressive modeling was applied assuming linear relationship between time and the outcome measures. Autoregressive models specify that the output variable depends linearly on its own previous values. Thus, an autoregressive model of order 1 means that a certain value depends on the value of the previous month, and autoregressive of order 2 means that depends on the values of the two previous months. In the final regression, we generated three models; non-autoregressive, autoregressive order 1 and 2 (see details in Additional file 1: Tables S1 and S2).

First, the period between January 2015 and March 2020 was modeled considering period before COVID-19 onset. As the exact point in time is not known when the centralized procurement changed the pattern of distribution, the break date was determined from the January 2015 to March 2020 period using the Supremum Wald test for a structural break at an unknown break date. Once determined, the break date in the first segment model the complete period (January 2015 to December 2020) was modeled introducing the change due to COVID-19 in April 2020. Considering the two possible changes, the model would be:

$${\text{Yt}} = \beta 0 + \beta {1} \times {\text{time}}\left( t \right) + \beta {2} \times {\text{intervention1}}\left( t \right) + \beta {3} \times {\text{time after intervention1}}\left( t \right) + \beta {4} \times {\text{intervention2}}\left( t \right) + \beta {5} \times {\text{time after intervention2}}\left( t \right) + \in \left( t \right).$$

The basic model included terms to estimate the intercept (*β*0), baseline trend (*β*1), changes in the level immediately after structural change (*β*2 and *β*4), and changes in the trend after the structural break (*β*3 and *β*5). The intervention 1 corresponds to the decentralized procurement estimated with the Supremum Wald test and intervention 2 corresponds to COVID-19. Time (*t*) is expressed in one-month intervals. To identify the most parsimonious models, backward elimination was used and excluded non-significant term (*p* > 0.05). The models were controlled for autocorrelation and seasonality. All analyses were carried out with STATA 17 (StataCorp, TX, US).

## Results

### Utilization of losartan and metformin

**Fig. 1:**
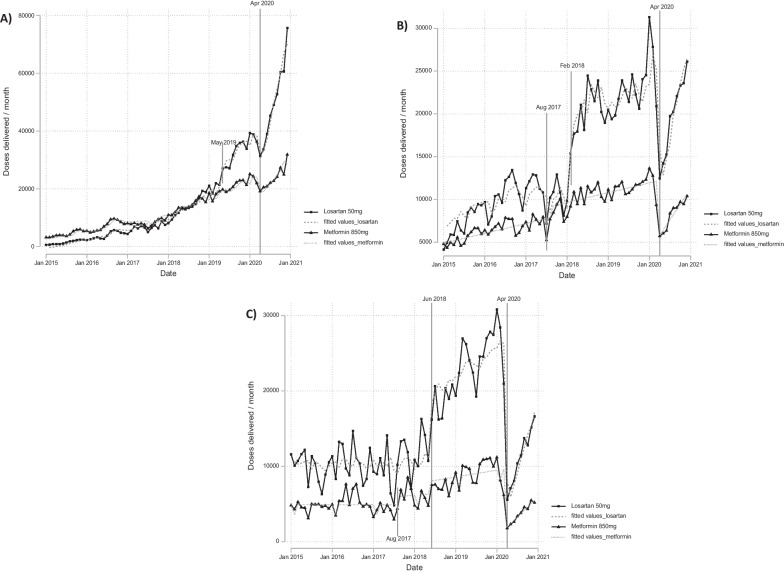
Quantity of doses delivered per month between January 2015 to December 2020 for Losartan 50 mg and Metformin 850 mg. **A** Primary level of care, **B** secondary level of care, and **C** tertiary level of care

In January 2015, the registered doses, one dose is equivalent to a standard monthly treatment, dispensed were around 200, 5000 and 6500 in the month, respectively, for the primary, secondary and tertiary level of care, for both losartan and metformin. We found in the primary level of care there was a monthly increase in doses dispensed over time, as shown in Fig. 1A. For losartan, there was a monthly increase of 152.39 doses during until April 2019, followed by a sudden increase in May 2019 to 5394.21 doses. Meaning that, from January 2015 to April 2019, in each month almost 152 more people nationwide received a monthly treatment of losartan, and suddenly in May 2019, almost 5394 people more than the baseline trend received the monthly treatment. For metformin, from January 2015 to March 2020, there was a steady increase in 92.61 doses dispensed each month.

In the secondary level of care (Fig. 1B), for losartan, there is no a clear monthly increase but a significant change in February 2018, where there was a jump with the increase of 6001.08 doses dispensed. While for metformin, there was a monthly increase in 95.43 doses from January 2015 to July 2017, then in August 2017 there was a sudden increase in the dispensing of 1317.00 doses. In the tertiary level of care, from 2015 to approximately 2017 a stationary trend over the mean for both losartan and metformin was observed (Fig. 1C). Then, for losartan there was also an increasing trend of 187.37 doses each month from June 2018 to March 2020. While for metformin in August 2017 there was a trend change with monthly increases of 33.72 doses, and in June 2018 another trend change occurred with monthly increases of 41.18 doses until March 2020. Additionally, we observed a seasonality pattern for metformin in the tertiary level as in all Februarys drops were observed of 1308.07 doses dispensed over the period study (Fig. 1C).

At all levels of care the COVID-19 onset caused abrupt drops in doses dispensed. The primary level was most affected with a fall in 165,830.00 doses for losartan and 44,886.59 doses for metformin (see Fig. 1). From May 2020 a progressive increase was identified at all levels of care, although by the end of 2020 neither the second or tertiary level of care reached pre-pandemic levels (Fig. 1B and C).

### Availability of losartan and metformin

**Fig. 2:**
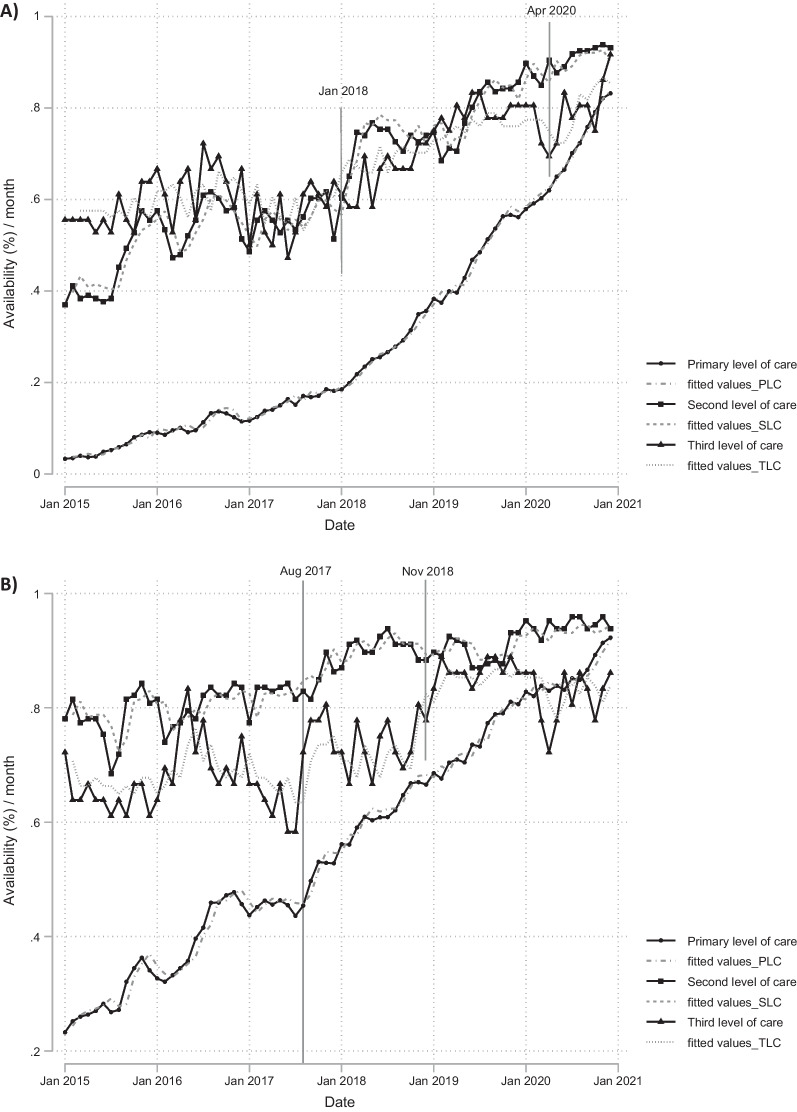
Availability per month between January 2015 and December 2020 in the all the levels of care. **A** Losartan 50 mg and **B** metformin 850 mg

The patterns of availability are similar for losartan and metformin (Fig. 2). In January 2015, availability of losartan was found to be 3%, 37% and 56%, for the primary, secondary, and tertiary level, respectively, while for metformin this was 23%, 78%, and 72%, respectively.

In the primary level of care there is a steady growth was observed over the period (2015–2020) for both losartan and metformin, but only for metformin there was an immediate increase of 2% in the availability after August 2017. There is no a clear baseline trend in the availability for the second and third level of care for both medicines. For the availability of metformin, an immediate increase of 4% was observed after August 2017 and of 7% after November 2018 in the secondary and tertiary level, respectively (Fig. 2A).

For availability of losartan at the secondary level, there is an increase of 5% immediately after January 2018. In the tertiary level, there is an increase of 7% immediately after January 2018 plus a decrease of 110% immediately after April 2020 followed by a growing increase of 2% per month (Table 2b). In addition, patterns of seasonality downward were detected on December for the primary and second level of care, with values of 1% and 3%, respectively (Fig. 2B).

## Discussion

### Key findings

In general, in plotting the use and availability of losartan and metformin, we observed an increasing trend with some changes at different points in time. Structural changes with significant impact on losartan dispensing occurred in the first semester of 2018 while for metformin this was in August of 2017 but also in June 2018. In April 2020, in spite of the availability in most of the facilities, COVID-19 caused a significant fall in medicines delivered at all levels of care affecting primary level facilities to a greater extent where 165,830 doses of losartan and 44,886 doses of metformin stopped delivering.

### Interpretation of results

There is no clear relationship between the changes detected over time with regard to utilization and availability of the medicines and the change of procurement type nor other policies on medicines or NCDs. Among the initiatives to promote diagnosis and treatment of NCDs in Peru over the past 5 years, there have been the development and implementation of clinical practice guidelines, training of health care professionals, centralized purchases for essential medicines [27, 28, 31, 32]. As the pattern of medicines utilization seems to grow over time, this range of different activities and other policies are needed in order to provide proper access to medicine for all in need.

Also, it is mandatory to rethink the procurement and supply chain management systems to respond to humanitarian crises like outbreaks, natural disasters, or political conflicts since the lack of contingency plans may generate disruption in the access to medicines as was observed with the onset of COVID-19. In Peru, it was not until July 2020 that the MoH published guidelines for the continuation of care of patients with NCDs during the COVID-19 pandemic [33]. These guidelines included home delivery of medicines, refill for more than one month, authorization for close relatives to pick up medications, and phone calls to monitor consultation and medicines needs. These actions were implemented in August onwards which may explain the increase trend in doses dispensed during that period. However, the failure to reach pre-pandemic levels are a reflection that these actions were not sufficient to meet people’s needs.

Even though the pattern of availability in the primary level of care has increased over time, efforts must be redoubled to enhance the services delivery at this level of care to not overcrowd other levels where better availability is observed. It has been reported that people prefer to seek care at a secondary and tertiary level of care because of high turnover of health care personnel, lack of specialist to manage NCDs at the primary level of care and insufficient resources (medicines, laboratory testing, etc.) [18, 20]. In addition, there is a feeling of mistrust by the population in the health system capacity. Peru has national clinical guidelines for the management of HTN and T2D in the primary level of care, but for proper implementation, the government needs to work on improving organizational, infrastructure and financial issues [34]. The MoH and government should implement strategies to facilitate the compliance with existing clinical guidelines and guarantee equitable access to medicine.

On the assumption of a scenario of inadequate programming and/or supply interruption, the high demand of medicines may cause a shortage that would drive to out-of-pocket expenses and compromise the adherence to treatment needed to achieve the desired clinical outcomes. Thus, health systems must ensure timely forecasting, purchasing, and distribution of essential medicines learning from previous experiences such as those introduced for the management of Human Immunodeficiency Virus (HIV) or infectious diseases [35]. Also, different types of follow-up enquiry are required in each case to identify bottlenecks and propose solutions.

In this study, we found there were heterogeneous breaks in time for medicines’ use and availability between the levels of care except for the COVID-19 onset that affect all levels. This may be attributed to the complexity of the interplay of pharmaceutical policy. For example, each facility at the tertiary level is financially independent for forecasting, purchasing and distributing medicines. In contrast, primary care facilities depend on regional health departments so any disruption in the supply chain can affect them. The interplay of pharmaceutical and health policies are also influenced by low public accountability and transparency, and high burden of governmental bureaucracy in LMICs [12].

Since medication for NCDs requires chronic use regardless the time of year, some patterns observed with our data found a seasonality effect. For utilization, in Februarys there is a decrease in the number of pills dispensed for metformin in the tertiary level of care and drops in all Decembers of losartan availability for the primary and secondary level of care. Although, we cannot attribute this to a proven factor, there are points that may help to explain this. For example, it is common for health care facilities to work on inventories and annual reports during Decembers and avoid high flows of incoming and outgoing medicines. Also, during the month of December, it is likely that people visit health facilities less because they spend time with their families for Christmas and New Year. Another aspect that may influence are summer holidays that occur during January and February where some families travel outside the city and do not frequent health care facilities.

### Limitations and strengths

Our study has some limitations and potential biases such as the definition of availability was built based on the dispensing of medicines during a month and it is likely to be overestimated as it is measured at one point at the end of the month and it does not take into account day-to-day variations. Also, SISMED’s reports are subject to occasional data entry errors which are not always checked at higher levels. Even if the medicines were considered available in this study, patients may have some problems finding them in the health care facilities or may have access them in the necessary quantity. However, given the number of patients with NCDs treated at a health care facility, the probability of no patient being treated in a month is low, which makes this indicator credible.

Additionally, given the insufficient data regarding the timing of the implementation of different strategies in each region, level and health care establishment, the impact of centralized procurement could not be tested of previously identified potentially relevant interventions and we present only structural changes identified during the analysis. Future evaluations should plan the collection of relevant information (such as time of implementation for each facility) to complement available information from SISMED and provide data to perform sub-analysis by regions.

In spite of the limitations, our study has used routine data on surveillance of dispensing and availability of essential medicines for NCDs that in contrast to other studies provide a snapshot of availability at the time of the study. The SISMED data are collected every month by all pharmacies in the country [36] and represent an important information source for relevant indicators related to access to care for patients living with NCDs. This data can be useful for the MoH and academia for monitoring and studies about medicines utilization. SISMED as a data source allows for a detailed understanding of within-country use and availability to inform decision-makers and planning interventions to address procurement and distribution issues as well as monitor their impact. Peru is a unique case of an LMIC with a country-level monitoring system that allows for stratified analysis. We strongly recommend the national authorities to periodically analyze information on medicine utilization and availability to: (1) evaluate the impact of policies conducted and their impact in different regions, levels of care and even individual sentinel health care establishments, and (2) identify any disruption in the supply chain of essential medicines.

## Conclusions

With the increasing prevalence of NCDs in Peru as well as in other countries globally sustainable and equitable access to essential medications is critical for the prevention of complications and early mortality. The availability of first-line medicines for HTN and T2D is an essential factor for this. As seen in this study, the continuous delivery of medicines for patients was not always ensured. During the first months of the COVID-19 pandemic, there was a significant fall in the dispensing of medicines, it is necessary to learn from this experience and develop plans to secure constant provision of medicines. The data collected by SISMED could potentially be used to track activities in different regions, levels of care and even health care establishments, as well as be used as a tool for monitoring changes in policy and practice. Similar tools for measuring availability and supply of medicines are necessary in other contexts to continuously monitor delivery and availability of medicines and the impact of policies, other changes in the health system and countrywide disruptions, such as COVID-19 or natural disasters, which might impact individuals needing to access their medicines.

## Supplementary Information

**Additional file 1: Table 1a.** Final time series model for quantity of doses dispensed per month of losartan 50mg over time. **Table 1b.** Final time series model for quantity of doses dispensed per month of metformin 850mg over time. **Table 2a.** Final time series model of availability per month of losartan 50mg over time. **Table 2b.** Final time series model of availability per month of metformin 850mg over time.

## Data Availability

Data for analysis are in the public domain. The data that support the finding of this study are available from the Peruvian National Regulatory Agency (Dirección General de Medicamentos Insumos y Drogas—DIGEMID, in Spanish), which provides a monthly updated repository of Availability of Pharmaceutical Products available in the following link: https://www.digemid.minsa.gob.pe/category/disponibilidad-productos-farmaceuticos
