# Supplementary material for: Using government data to understand the use and availability of medicines for hypertension and diabetes: lessons from Peru
Source: J Pharm Policy Pract. 2022 Nov 18;15:86. doi: 10.1186/s40545-022-00481-5 (PMC9675072; doi:10.1186/s40545-022-00481-5)
Supplement: Supplementary file 1 — Additional file 1: Table 1a. Final time series model for quantity of doses dispensed per month of losartan 50mg over time. Table 1b. Final time series model for quantity of doses dispensed per month of metformin 850mg over time. Table 2a. Final time series model of availability per month of losartan 50mg over time. Table 2b. Final time series model of availability per month of metformin 850mg over time. [file 40545_2022_481_MOESM1_ESM.docx]

| **Supplementary table 1a. Final time series model for quantity of doses dispensed per month of losartan 50mg over time** | | | | | | | | | | |
| --- | --- | --- | --- | --- | --- | --- | --- | --- | --- | --- |
|  |  |  |  |  |  |  |  |  |  |  |
|  | Primary level of care | | | Second level of care | | | Third level of care | | | |
|  | R-squared = 0.990 | | | R-squared = 0.908 | | | R-squared = 0.856 | | | |
|  | Coefficient | Standard error | p-value | Coefficient | Standard error | p-value | Coefficient | Standard error | p-value |  |
| Intercept | -1075.83 | 593.1 | 0.071 | 4730.68 | 820.32 | 0.001 | 7986.2 | 1067.08 | 0.001 |  |
| Baseline trend | 152.39 | 34.82 | 0.001 | - | - | - | - | - | - |  |
| Level change (February 2018) | - | - | - | 6001.08 | 1074.85 | 0.001 | - | - | - |  |
| Trend change (June 2018) | - | - | - | - | - | - | 187.37 | 26.04 | 0.001 |  |
| Level change (May 2019) | 5394.21 | 1318.71 | 0.001 | - | - | - | - | - | - |  |
| Level change (April 2020) | -165830.00 | 24203.33 | 0.001 | -78027.76 | 19080.07 | 0.001 | -87514.25 | 22616.34 | 0.001 |  |
| Trend change (April 2020) | 2532.35 | 364.15 | 0.001 | 1133.31 | 279.53 | 0.001 | 1107.83 | 331.80 | 0.001 |  |
| Autoregressive factor | 0.63 (L=1) | 0.08 | 0.001 | 0.51 (L=1) | 0.08 | 0.001 | 0.29 (L=1) | 0.10 | 0.020 |  |

| **Supplementary table 1b. Final time series model for quantity of doses dispensed per month of metformin 850mg over time** | | | | | | | | | | | | | | | | | | |  |
| --- | --- | --- | --- | --- | --- | --- | --- | --- | --- | --- | --- | --- | --- | --- | --- | --- | --- | --- | --- |
|  |  | |  | |  | |  | |  | |  | |  | |  | |  | |  |
|  | Primary level of care | | | | | | Second level of care | | | | | | Third level of care | | | | | |  |
|  | R-squared = 0.970 | | | | | | R-squared = 0.871 | | | | | | R-squared = 0.761 | | | | | |  |
|  | Coefficient | | Standard error | | p-value | | Coefficient | | Standard error | | p-value | | Coefficient | | Standard error | | p-value | |  |
| Intercept | 269.83 | | 343.92 | | 0.436 | | 4800.29 | | 259.17 | | 0.001 | | 4926.67 | | 208.71 | | 0.001 | |  |
| Baseline trend | 92.61 | | 29.19 | | 0.002 | | 95.43 | | 12.43 | | 0.001 | | - | | - | | - | |  |
| Level change (August 2017) | - | | - | | - | | 1317 | | 451.99 | | 0.005 | | - | | - | | - | |  |
| Trend change (August 2017) |  | |  | |  | |  | |  | |  | | 33.72 | | 11.79 | | 0.006 | |  |
| Trend change (June 2018) | - | | - | | - | | - | | - | | - | | 41.18 | | 11.55 | | 0.001 | |  |
| Level change (April 2020) | -44886.59 | | 11693.35 | | 0.001 | | -39149.93 | | 7902.37 | | 0.001 | | -32362.29 | | 10194.94 | | 0.002 | |  |
| Trend change (April 2020) | 668.60 | | 171.94 | | 0.001 | | 511.42 | | 116.44 | | 0.001 | | 383.51 | | 149.91 | | 0.013 | |  |
| Autoregressive factor | 0.73 (L=1) | | 0.08 | | 0.001 | | - | | - | | - | | - | | - | | - | |  |
| Seasonality (February) | - | | - | | - | | - | | - | | - | | -1308.07 | | 498.18 | | 0.011 | |  |
| **Supplementary Table 2a. Final time series model of availability per month of losartan 50mg over time** | | | | | | | | | | | | | | | | | | | |
|  | |  | |  | |  | |  | |  | |  | |  | |  | |  | |
|  | | Primary level of care | | | | | | Second level of care | | | | | | Third level of care | | | | | |
|  | | R-squared = 0.998 | | | | | | R-squared = 0.963 | | | | | | R-squared = 0.717 | | | | | |
|  | | Coefficient | | Standard error | | p-value | | Coefficient | | Standard error | | p-value | | Coefficient | | Standard error | | p-value | |
| Intercept | | 0.003 | | 0.002 | | 0.091 | | 0.078 | | 0.023 | | 0.001 | | 0.286 | | 0.06 | | 0.001 | |
| Level change (January 2018) | | - | | - | | - | | 0.046 | | 0.014 | | 0.002 | | 0.069 | | 0.02 | | 0.001 | |
| Level change (April 2020) | | - | | - | | - | | - | | - | | - | | -1.100 | | 0.507 | | 0.034 | |
| Trend change (April 2020) | | - | | - | | - | | - | | - | | - | | 0.017 | | 0.007 | | 0.028 | |
| Autoregressive factor | | 1.032 (L=1) | | 0.005 | | 0.001 | | 0.862 (L=1) | | 0.044 | | 0.001 | | 0.520 (L=2) | | 0.101 | | 0.001 | |
| Seasonality (December) | | -0.0136 | | 0.004 | | 0.002 | | -0.031 | | 0.014 | | 0.031 | | - | | - | | - | |

| **Supplementary Table 2b. Final time series model of availability per month of metformin 850mg over time** | | | | | | | | | |
| --- | --- | --- | --- | --- | --- | --- | --- | --- | --- |
|  |  |  |  |  |  |  |  |  |  |
|  | Primary level of care | | | Second level of care | | | Third level of care | | |
|  | R-squared = 0.998 | | | R-squared = 0.8390 | | | R-squared = 0.7365 | | |
|  | Coefficient | Standard error | p-value | Coefficient | Standard error | p-value | Coefficient | Standard error | p-value |
| Intercept | 0.019 | 0.007 | 0.008 | 0.256 | 0.066 | 0.001 | 0.331 | 0.069 | 0.001 |
| Level change (August 2017) | 0.016 | 0.007 | 0.021 | 0.036 | 0.011 | 0.002 | - | - | - |
| Level change (November 2018) | - | - | - | - | - | - | 0.074 | 0.018 | 0.001 |
| Autoregressive factor | 0.966 (L=1) | 0.017 | 0.001 | 0.681 (L=1) | 0.083 | 0.001 | 0.520 (L=1) | 0.100 | 0.001 |
